# Hemifield Effects in Multiple Identity Tracking

**DOI:** 10.1371/journal.pone.0043796

**Published:** 2012-08-28

**Authors:** Charlotte Hudson, Piers D. L. Howe, Daniel R. Little

**Affiliations:** Melbourne School of Psychological Sciences, The University of Melbourne, Parkville, Victoria, Australia; Macquarie University, Australia

## Abstract

In everyday life, we often need to attentively track moving objects. A previous study has claimed that this tracking occurs independently in the left and right visual hemifields (Alvarez & Cavanagh, 2005, *Psychological Science,16*, 637–647). Specifically, it was shown that observers were much more accurate at tracking objects that were spread over both visual hemifields as opposed to when all were confined to a single visual hemifield. In that study, observers were not required to remember the identities of the objects. Conversely, in real life, there is seldom any benefit to tracking an object unless you can also recall its identity. It has been predicted that when observers are required to remember the identities of the tracked objects a bilateral advantage should no longer be observed (Oksama & Hyönä, 2008, *Cognitive Psychology, 56*, 237–283). We tested this prediction and found that a bilateral advantage still occurred, though it was not as strong as when observers were not required to remember the identities of the targets. Even in the later case we found that tracking was not completely independent in the two visual hemifields. We present a combined model of multiple object tracking and multiple identity tracking that can explain our data.

## Introduction

Successful interaction with one’s environment requires the ability to pay attention to objects of interest. As objects are frequently in motion, tracking is often required to maintain attention on them as their locations change. Accordingly, object tracking is involved in numerous everyday activities. When waiting to cross the street, for instance, it is necessary to track cars and bicycles to judge a safe time to step out onto the road.

To explore the cognitive processes involved in object tracking in a laboratory setting, Pylyshyn and Storm [1] developed the multiple object tracking (MOT) paradigm. The task requires observers to track a subset of identical objects that were briefly cued as targets while they move on a computer monitor. Target tracking accuracy is measured either by asking the observer to click on all the targets at the end of the trial (e.g. [2], [3]) or by asking the observer whether a randomly selected object was a target (e.g. [4], [5]). The MOT paradigm has been a popular choice among researchers as a way of studying the deployment of attention in dynamic scenes [6].

One of the most salient findings from the MOT literature has been that object tracking is highly capacity limited. People are limited to tracking approximately three to five objects [1], [5], [7], [8]. Individual differences, however, have been reported [9], with expertise evident in people whose occupations rely on advanced spatial cognitive abilities, such as radar operators [10], [11]. It has also been suggested that the capacity limit is context-dependent, with factors such as the objects’ movement speed ([12] cf. [13]) and object crowding [14] affecting tracking ability.

In a seminal MOT study, Alvarez and Cavanagh [4] directly investigated the source of the object tracking capacity limitation. Given that early visual processing occurs almost exclusively in the cerebral hemisphere contralateral to the visual hemifield of input [15], Alvarez and Cavanagh investigated whether there would be independent constraints for tracking objects in the left and right visual hemifields. To examine this, they modified the typical MOT display by partitioning it into quadrants and adding a central fixation cross. In the baseline condition, observers tracked two targets, both confined to a single hemifield. In the unilateral condition observers tracked four targets, all confined to one visual hemifield. In the bilateral condition, the observers again tracked four targets, but the targets were spread across both hemifields. Their results indicated equal tracking accuracy in the baseline and bilateral conditions, but a dramatic drop in performance in the unilateral condition.

Alvarez and Cavanagh’s [4] finding supported the existence of two separate tracking mechanisms, one in the right cerebral hemisphere responsible for tracking objects in the left visual hemifield and another in the left cerebral hemisphere responsible for the right visual hemifield. Both mechanisms could track two, but not four, targets. Thus, observers were highly accurate in the baseline and bilateral conditions because in both conditions there were never more than two targets in a single hemifield. Conversely, observers were overwhelmed by the unilateral condition, as this required them to attempt to track four targets in a single hemifield.

Despite the importance of Alvarez and Cavanagh’s [4] results, the MOT paradigm is limited in its applicability because all the objects are identical [9], [16]. It is challenging to imagine an everyday scenario in which one is required to track a subset of indistinguishable items. Rather, we tend to experience situations in which the objects we need to track have unique identities that we are required to remember [17]. The advent of multiple identity tracking (MIT), as coined by Oksama and Hyönä [9], acknowledges this. MIT tasks are a modified version of MOT, typically using easily distinguishable objects that remain visible for the duration of movement and are then masked at the end of the trial (e.g. [16]–[19]).

While this might initially seem like a trivial distinction, MIT may entail fundamentally different processing to MOT. While both tasks require objects to be tracked as their locations change, MIT additionally requires objects’ unique identities to be bound to their locations. Thus, the observer must solve the ‘binding problem’ [20]. Maintenance of identity-location bindings has been conceptualised as an effortful process that requires sustained attention [21]. This has led some authors to conclude that two separate tracking systems are required, one for identities and another for locations [16], while others have found evidence in support of a common-resource model [17], [22].

Crucially, the mechanism that solves the binding problem has classically been considered a *serial* mechanism, meaning that each object must be attended to sequentially to refresh its identity-location binding [20], [23]. Indeed, the only model of MIT to date, the Oksama and Hyönä [24] model of multiple identity tracking (MOMIT), posits that tracking unique objects cannot occur in parallel. Oksama and Hyönä argue that binding identity to location is a demanding task that can only be achieved via a serial switching mechanism that cycles through the tracked targets, attending to each one in turn. The identity-location bindings are held in an episodic buffer and each binding deteriorates if attention takes too long to return to a target.

MOMIT [24] accordingly predicts that the bilateral advantage observed by Alvarez and Cavanagh [4] when observers were required to track identical objects (i.e. MOT) will not exist when observers are required to track unique objects (i.e. MIT) because it is indicative of parallel processing. The model proposes that for MIT, tracking performance is limited by a single resource that is shared between the two hemispheres. Thus, there should be no difference in tracking performance regardless of whether all the targets are located within one hemifield or divided between two hemifields. Although this is a bold prediction, it accords with Alvarez and Cavanagh’s suggestion ([4], p. 642) that hemifield independence would not occur in tasks that involve attending to identity information, because such processes are mediated by brain areas further along the visual processing pathway, where information from the two visual hemifields is not separated. The MOMIT prediction is also consistent with the findings of a related change-detection study by Delvenne [25]. When observers performed a memory change-detection task that did not involve identity-location bindings, a bilateral hemifield advantage was observed. No such advantage was found, however, when observers performed another memory change-detection task that did involve identity-location bindings.

The primary aim of the present study was to test the prediction of MOMIT [24] that the hemifield effect reported by Alvarez and Cavanagh [4] when observers performed MOT should not occur when observers perform MIT. To preview our results: The following four experiments demonstrated a reduction of the hemifield effect in MIT relative to MOT. Crucially, even when performing MIT a robust hemifield effect is still observed, contrary to previous findings [4]. The theoretical implications of these findings are reviewed in the Discussion.

## Experiment 1A

In this experiment, we adapted Alvarez and Cavanagh’s [4] MOT paradigm to a typical MIT display, in which the unique identities of objects were visible for the duration of the movement phase. As in the previous study, there were three conditions: a baseline condition in which observers tracked two targets, both located in the same quadrant; a bilateral condition in which there were four targets in total, two in each visual hemifield; and a unilateral condition in which four targets were confined to just one hemifield. MOMIT [24] predicts that a) tracking accuracy will be higher in the baseline condition than in the other two conditions because it requires fewer targets to be tracked, and b) tracking accuracy for the bilateral and unilateral conditions will be equal.

### Method

#### Participants

Twenty-eight participants (24 female) aged 19–34 with normal or corrected-to-normal vision completed the experiment. All observers provided informed written consent and the study was approved by the Department Human Ethics Advisory Group in the School of Psychological Sciences at the University of Melbourne. Observers were reimbursed $15 for their time.

#### Equipment

Participants had normal or corrected-to-normal visual acuity as tested by a Good-Lite near vision chart at a distance of 40 cm. Stimuli were presented on a 21′′ Sony or Dell CRT monitor at a resolution of 1280×1024 with a refresh rate of 85 Hz. Observers viewed the stimuli from a distance of 60 cm. The experiment was run on a Windows XP operating system using Psychophysics Toolbox routines (version 3 [26], [27]) for MATLAB (Natick, MA, USA). Participants completed the experiment individually in a small testing room with the lights off.

#### Stimuli

**Figure 1:**
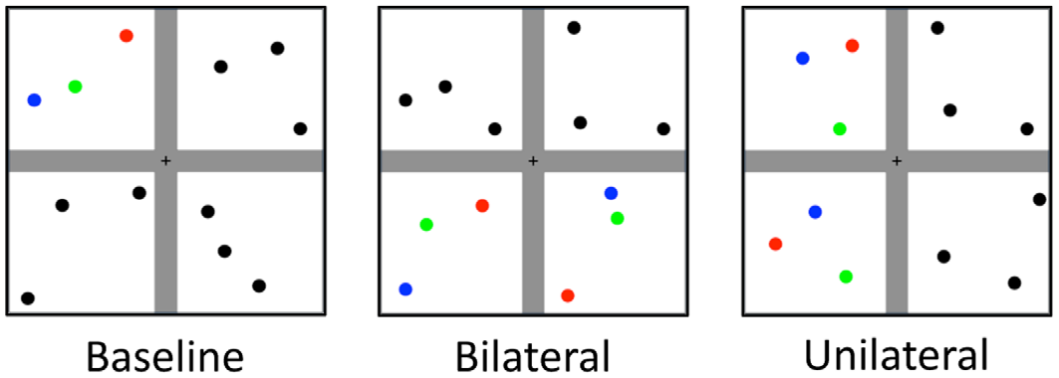
Schematic depiction of the three trial conditions for Experiment 1. Targets are divided between both visual hemifields on bilateral trials, and confined to one hemifield on unilateral trials.

The stimuli were adapted from Alvarez and Cavanagh [4]. Observers saw a white square display that subtended 23°×23° of visual angle. There was a central black fixation cross that subtended 0.48°×0.48°. Two thick grey lines with a subtended width of 3°, one aligned vertically and the other horizontally, divided the display into four equal quadrants that each subtended 10°×10°. Moving disks with subtended diameters of 0.76° occupied each quadrant. The maximum distance a disk could travel from fixation was 15.8°. Each disk moved in straight lines except when bouncing after reaching a border or coming within 2° of another disk. Each quadrant contained three disks (Figure 1). In the baseline condition, the disks in one quadrant were coloured. In the other two conditions, the disks in two quadrants were coloured. These two quadrants were arranged either bilaterally or unilaterally.

### Procedure

Participants were instructed to fixate on the central cross and track the coloured disks. Participants were instructed to employ a strategy of tracking two targets in a quadrant because the identity of the third could then be deduced. This was done to ensure that all participants used the same tracking strategy.

The targets’ colours remained visible while the disks moved within their quadrant for eight seconds. The disks then stopped moving and were concealed by a multi-color square mask for 50 ms before reappearing in black. One of the target disks was then shown in red, green, or blue. Participants responded to the question “Was this colour target at this position?" using the ‘y’ key for yes and the ‘n’ key for no. This process was repeated for a second target from the same quadrant. If an error was made, the message “[one/two] mistake[s]" was displayed at the end of the trial.

The experiment began with five practice trials. A 50 trial QUEST staircase routine followed [28], [29], which found the disk speed at which each participant could achieve 75% tracking accuracy in the baseline condition. This allowed floor and ceiling effects to be avoided and controlled for individual differences in tracking ability [9]. Each participant’s unique speed was used for the proceeding 240 experimental trials, which comprised equal numbers of baseline, bilateral and unilateral trials randomly interleaved. Thus, for each of the three conditions 80 trials were run and the average accuracy was calculated.

### Results & Discussion

Three participants had to be excluded due to a programming error that invalidated their data. Data for the remaining 25 participants was analysed. A unique speed was generated for each individual participant using the QUEST routine (mean speed = 12.1°/s).

**Figure 2:**
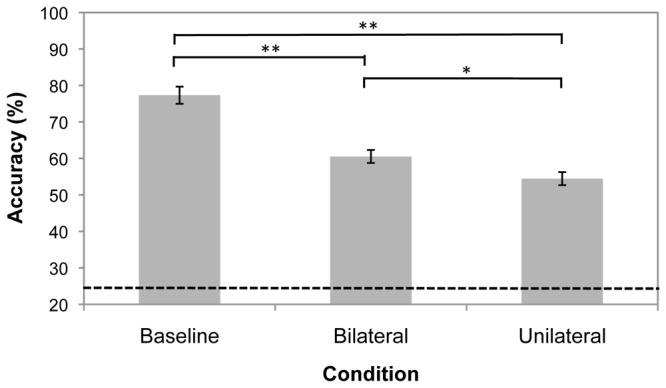
Mean tracking accuracy for the three conditions in Experiment 1. The broken line shows chance performance, which is 25% as two yes/no questions were asked per trial and both had to be answered correctly for the trial to be counted as correct. Error bars denote within-observers standard error [38]. *Significantly different at *p*<0.01; **Significantly different at *p*<0.001.

Figure 2 depicts our results. A repeated measures Analysis of Variance (ANOVA) with sphericity assumed (Mauchly’s test *p* = 0.15) indicated a significant main effect of condition (*F*(2, 48) = 53.4, *p*<0.001, partial *η^2^* = 0.69). As expected, there was an effect of set size, with tracking performance decreasing significantly in the bilateral and unilateral conditions compared to the baseline condition (*t*(24) = 7.00, *p*<0.001; *t*(24) = 9.12, *p*<0.001, respectively). Contrary to the prediction of MOMIT, a hemifield effect was evident: tracking accuracy was higher when targets were arranged bilaterally compared to unilaterally (*t*(24) = 3.40, *p* = 0.002). Bonferroni corrections were not applied here or in the other experiments of this article because they are not needed provided three or fewer groups are being compared and an ANOVA has already demonstrated a main effect [30].

At odds with the serial, non-hemifield-specific tracking mechanism predicted by MOMIT [24], our results indicate the existence of a bilateral advantage for tracking unique objects. It is possible that this unexpected hemifield effect was an artifact of participants mis-fixating vertically on bilateral trials, which would decrease the eccentricity of the objects and make the task easier. We addressed this concern by repeating the experiment with the addition of an Arrington Research eye tracker to monitor participants’ gaze on the fixation cross.

## Experiment 1B

This experiment was a replication of the previous experiment using an eye tracker. Eight participants (two female; seven of them new, including author P.H.) aged 21–35 were recruited in the manner described above. The message ‘fixation broken’ was displayed whenever the observers failed to maintain fixation on the fixation cross, after which the trial was restarted.

As before, the QUEST staircase routine produced a unique speed for each participant (mean speed = 21.7°/s), which ensured that accuracy in the baseline condition was approximately 75%. Mean accuracies were very similar to before: 72%, 57%, and 51% for the baseline, bilateral and unilateral conditions, respectively (for the previous experiment the corresponding accuracies were 77%, 61% and 54%). Although the overall accuracies were slightly lower when the eye-tracker was used, the relative accuracies were very similar. Crucially, the difference between the bilateral and the unilateral conditions was not significantly different between the two experiments (*t*(31) = 0.106, *p* = 0.92).

This replication using an eye-tracker rules out the possibility that the bilateral advantage we observed in Experiment 1a was due to participants mis-fixating vertically on bilateral trials. Perhaps then the hemifield effect is a manifestation of participants ‘cheating’ rather than actively tracking? We addressed this possibility in Experiment 2.

## Experiment 2

As the targets’ identities were continuously visible in Experiment 1, perhaps sustained tracking was not actually required: it is possible that participants only needed to attend to the targets near the end of the trial to see their colour and final location. We addressed this possibility by repeating our previous experiment with one alteration. This time, we only briefly cued the targets’ unique identities at the start of the trial. After this point, all the disks turned black and were visually indistinguishable. This forced participants to attentively track the targets for the entire trial, as in a standard MOT task.

### Method

#### Participants

Thirty-one people (20 female) ranging in age from 17–29 with normal or corrected-to-normal vision took part. Twenty-six of these participants were first year undergraduate psychology students from the University of Melbourne who received course credit for their participation; the remainder were personal contacts of author C.H. who did not receive reimbursement. As before, all observers provided informed written consent and the study was approved by the Department Human Ethics Advisory Group in the School of Psychological Sciences at the University of Melbourne.

#### Stimuli & Procedure

Very similar stimuli to Experiment 1 were used. As in Experiment 1 the targets moved within their quadrant for eight seconds. For the first three seconds the targets were coloured red, green, and blue; for the remaining five seconds all were black. Participants responded in the same way as Experiment 1, and the same trial structure was used (10 practice trials; 50 trial Quest routine; 240 experimental trials).

### Results & Discussion

Data for two participants was excluded because it was incomplete and two other participants’ data was excluded because a failure of the QUEST routine caused their baseline accuracy to be too high (>95%) thereby causing a ceiling effect. Data for the remaining 27 participants was analysed. The QUEST procedure allocated a unique speed to each participant (mean = 4.69°/s).

**Figure 3:**
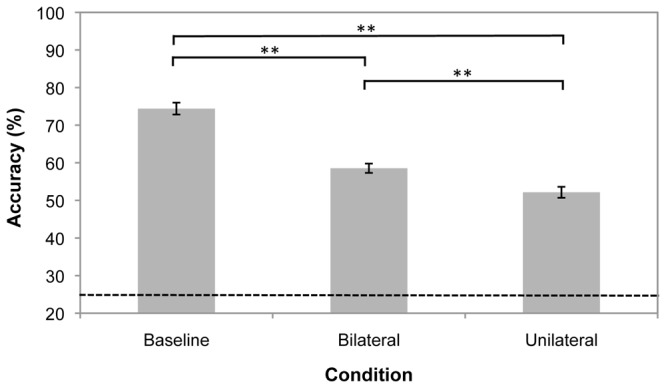
Mean tracking accuracy for the three conditions in Experiment 2. The broken line indicates chance performance is 25%. Error bars are within-observers standard error. **Significantly different at *p*<0.001.

Results are shown in Figure 3. A repeated-measures ANOVA with sphericity assumed (Mauchly’s test *p* = 0.38) indicated a main effect of condition (*F*(2, 52) = 95.9, *p*<0.001, partial *η^2^* = 0.79). Tracking accuracy was significantly higher in baseline trials than bilateral trials (*t*(26) = 9.78, *p*<0.001) and unilateral trials (*t*(26) = 12.0, *p*<0.001). There was also a hemifield effect evident, with bilateral tracking accuracy significantly greater than unilateral tracking accuracy (*t*(26) = 4.36, *p*<0.001).

We compared the results of Experiment 2 to those of Experiment 1a. A mixed 3 × 2 ANOVA with sphericity assumed (Mauchly’s test *p* = 0.06) indicated a significant main effect of condition (*F*(2, 100) = 138, *p*<0.001, partial *η^2^* = 0.74) but no significant difference between the two experiments (*F*(1, 50) = 0.43, *p* = 0.51, partial *η^2^* = 0.01). There was no significant interaction between condition and experiment (*F*(2, 100) = 0.06, *p* = 0.95, partial *η^2^*<0.01) indicating that tracking accuracy differed based on trial type, but not due to target identities being visible versus concealed.

This experiment replicates our findings from Experiment 1a and rules out the prospect that the bilateral advantage was merely a reflection of sustained tracking not being required due to the target identities being continuously visible. Accordingly, the data from both our experiments are incongruent with MOMIT [24].

One might therefore expect our data to replicate the Alvarez and Cavanagh [4] finding of independent tracking in the left and right visual hemifields. Specifically, they found that observers were as accurate at simultaneously tracking two targets in each hemifield (a total of four targets) as they were when just tracking two targets. Our data from Experiments 1 and 2 do not fit Alvarez and Cavanagh’s predictions as our observers were significantly worse at tracking four targets than two. There are two important differences between our stimuli and those used by Alvarez and Cavanagh that may account for this discrepancy. First, we used an MIT task whereas Alvarez and Cavanagh used an MOT task. Second, in our stimuli there were three disks in each quadrant whereas in their stimuli there were four disks in each quadrant. In Experiment 3 we will investigate the latter difference first by altering the number of disks to more closely resemble Alvarez and Cavanagh’s original display.

## Experiment 3

In the Alvarez and Cavanagh [4] experiment target quadrants held four disks; two targets and two distractors. When we adapted their display to MIT in Experiment 1 and 2, we used three unique disks in the target quadrants. We presumed this would be equivalent under the rationale that only two disks would need to be tracked as the identity of the third could then be deduced. In Experiment 3 we controlled for the possibility that this assumption may have been incorrect. We altered our display so that it had the same number of disks in target quadrants as Alvarez and Cavanagh’s experiment: two unique targets and two distractors.

### Method

#### Participants

Twenty-five people (17 female) aged 18–30 with normal or corrected-to-normal visual acuity took part. One participant was a personal contact of author C.H.; the others were recruited via posters in the University of Melbourne psychology building and were reimbursed $15. As before, all observers provided informed written consent and the study was approved by the Department Human Ethics Advisory Group in the School of Psychological Sciences at the University of Melbourne.

#### Stimuli & Procedure

Very similar stimuli and procedure to the previous experiments were used, except that in each quadrant there were four disks. In the quadrants that contained targets, the two target disks were briefly coloured and the two distractor disks were always black. As before, targets were coloured red, green, or blue, and targets in the same quadrant were never the same colour as one another. The disks in the other quadrants were all black. As in Experiment 2, the targets’ colours were shown only for the first three seconds of the trial, after which they turned black and became indistinguishable from each other and the distractors. The same response method and trial structure as Experiment 2 was used.

### Results & Discussion

Due to a problem with the QUEST staircase procedure, one participant’s baseline accuracy was greater than 95%, so this participant was excluded because of ceiling effects. Data for the remaining 24 participants was analysed. The QUEST procedure allocated a speed for each observer (mean = 5.34°/s).

**Figure 4:**
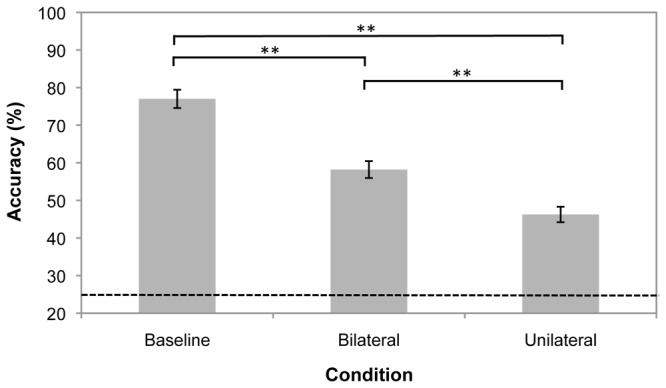
Mean tracking accuracy for the three conditions in Experiment 3. Broken line shows chance performance is 25%. Error bars are within-observers standard error. **Significantly different at *p*<0.001.

Results are shown in Figure 4. A repeated measures ANOVA with sphericity assumed (Mauchly’s test *p* = 0.68) indicated a main effect of condition (*F*(2, 46) = 71.0, *p*<0.001, partial *η^2^* = 0.76). There was an effect of set size, with tracking accuracy greater for baseline trials than bilateral trials (*t*(23) = 6.72, *p*<0.001) as well as unilateral trials (*t*(23) = 11.8, *p*<0.001). There was also a hemifield effect, with tracking accuracy significantly higher for bilateral relative to unilateral trials (*t*(23) = 5.01, *p*<0.001).

We compared our results to those of Experiment 2. A mixed 3×2 ANOVA with sphericity assumed (Mauchly’s test *p* = 0.46) showed a significant main effect of condition (*F*(2, 98) = 159, *p*<0.001, partial *η^2^* = 0.77); no significant main effect of experiment (*F*(1, 49) = 0.13, *p* = 0.72, partial *η^2^* = 0.96); and a significant interaction between condition and experiment (*F* (2, 98) = 4.10, *p* = 0.02, partial *η^2^* = 0.08). Independent t-tests, however, indicated that there were no significant differences between Experiment 2 and 3 on baseline (*t* (49) =  −0.73, *p* = 0.47); bilateral (*t* (49) = 0.09, *p* = 0.93); or unilateral trials (*t* (49) = 1.66, *p* = 0.10).

This experiment replicates the findings of our previous two experiments. We have robustly demonstrated a bilateral tracking advantage for uniquely identifiable targets. Observers found it easier to track four targets when they are spread between two hemifields than confined to a single hemifield. We failed to demonstrate, however, that tracking is independent in the two hemifields. Observers found it easier to track two targets in a single hemifield (i.e. the baseline condition) than to simultaneously track two targets in each hemifield, suggesting in the former case they were able to utilise resources from both hemifields. This latter finding is not in agreement with Alvarez and Cavanagh [4]. To determine whether the contrast between Alvarez and Cavanagh’s results and our own was truly due to our targets having unique identities, we attempted to replicate their original MOT study in Experiment 4.

## Experiment 4

In an MIT experiment each target has an unique identity and so at the end of the trial the observer is asked to identify a *particular* target, e.g. the red one. Conversely, in MOT, all the targets are identically coloured and at the end of the trial the observer merely has to identify the location of the targets, without having to recall their identities. Thus, MOT does not require identity-location bindings.

In this experiment we attempted to replicate the Alvarez and Cavanagh’s [4] MOT study. If our previous three experiments are genuinely displaying something specific to MIT, then we should get contrasting results here. If Alvarez and Cavanagh’s conclusion is correct, when using an MOT stimulus we would expect to see equal performance in the baseline and bilateral trials, and a large decrement in unilateral tracking relative to the other two conditions. Alternatively, if our previous experiments are simply demonstrating something generic to object tracking, we would expect the results of this experiment to be identical to those of Experiment 3.

### Method

#### Participants

Twenty-nine people (21 female) aged 17–33 with normal or corrected-to-normal visual acuity took part. Twenty-six participants were University of Melbourne first year undergraduate psychology students recruited via posters who received course credit; the other three participants were personal contacts of author C.H. who did not receive reimbursement. As before, all observers provided informed written consent and the study was approved by the Department Human Ethics Advisory Group in the School of Psychological Sciences at the University of Melbourne.

#### Stimuli & Procedure

Unlike the previous experiments in which observers performed MIT, in this experiment observers performed MOT. As in Experiment 3 each quadrant always held four disks. In the quadrants that contained targets, two disks were identified as targets by briefly turning the *same* colour (either red, green, or blue) at the start of the trial. At the end of the trial, two disks from the same target quadrant were highlighted, one at a time, and the observers were asked whether or not they were targets. As no distinction was made between targets (since they were always the same colour as each other) this experiment did not require colour to be bound to location, and was thus a pure MOT experiment.

### Results & Discussion

The data for three participants had to be excluded due to a computer error that corrupted their data. Data for the remaining 26 participants was analysed. The QUEST routine produced unique speeds for each participant (mean = 4.39°/s).

**Figure 5:**
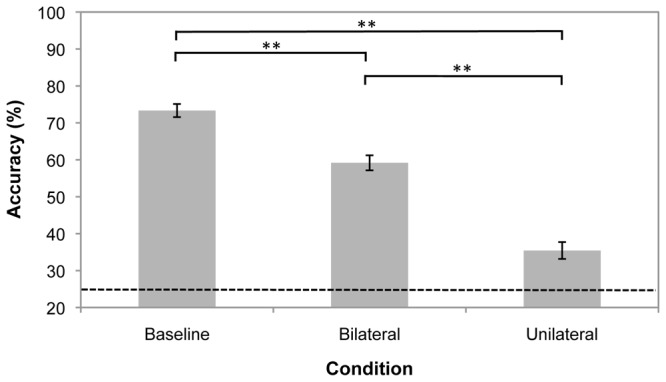
Mean tracking accuracy for the three conditions in Experiment 4. The broken line shows that chance performance is 25%. Error bars are within-observers standard error. **Significantly different at *p*<0.001.

Results are shown in Figure 5. A univariate ANOVA with sphericity assumed (Mauchly’s test *p* = 0.35) showed a main effect of condition (*F*(2, 50) = 132, *p*<0.001, partial *η^2^* = 0.84). As expected, there was a hemifield effect, with bilateral tracking accuracy significantly greater than unilateral tracking accuracy (*t*(25) = 9.03, *p*<0.001). In contrast to the Alvarez and Cavanagh [4] study, baseline accuracy was significantly higher than accuracy in the bilateral condition (*t*(25) = 6.94, *p*<0.001).

Baseline and bilateral tracking accuracy looked similar to Experiment 3, but unilateral accuracy appeared greatly reduced. We compared Experiment 4 to Experiment 3 using a mixed 3×2 ANOVA with sphericity assumed (Mauchly’s test *p* = 0.95). We found a significant main effect of condition (*F*(2, 96) = 192, *p*<0.001, partial *η^2^* = 0.80); no main effect of experiment (*F* (1, 48) = 2.29, *p* = 0.14, partial *η^2^* = 0.05), and a significant interaction between condition and experiment (*F* (2, 96) = 5.76, *p* = 0.004, partial *η^2^* = 0.12). Between Experiment 3 and 4 there was no significant difference in baseline (*t*(48) = 1.09, *p* = 0.28) or bilateral (*t*(48) =  −0.23, *p* = 0.82) trials. Tracking performance on unilateral trials was significantly lower in Experiment 4 than in Experiment 3 (*t*(48) = 3.90, *p*<0.001).

As in our previous three experiments, we have found a set size effect with bilateral tracking accuracy being lower than baseline, indicating a capacity limitation. This is in contrast to Alvarez and Cavanagh’s [4] findings, in which four targets could be tracked as well as two, provided the four targets were displayed bilaterally. In agreement with Alvarez and Cavanagh’s results, we found a large drop in accuracy when targets were confined to a single hemifield, relative to when they were divided between both hemifields. This hemifield effect is more pronounced than in our previous three MIT experiments.

In the Alvarez and Cavanagh [4] experiment, when each trial ended, the observer was asked a single question: one disk was highlighted and the observer was asked whether or not it was a target. Conversely, in our Experiment 4, at the end of the trial the observer was asked two questions: two disks were highlighted in succession and the observer was asked whether both of these disks were targets. A trial was only counted as correct if observers correctly answered *both* questions. Accordingly, by taking the square root of our mean accuracy data, we can find the equivalent probability of getting one question per trial correct, facilitating a direct comparison of our data to that of Alvarez and Cavanagh. The probability of being able to answer a single question correctly in our Experiment 4 was 86% for the baseline condition, 77% for the bilateral condition, and 60% for the unilateral condition. Overall accuracy was slightly higher in the Alvarez and Cavanagh study, presumably because their disks were slightly larger than ours and their disk density was slightly less. Both factors would tend to increase tracking accuracy [31]. Their accuracies for the baseline, bilateral and unilateral conditions were 92%, 87%, and 66%, respectively. The difference in accuracy between the baseline condition and the bilateral condition in Alvarez and Cavanagh’s study, however, was similar to that in Experiment 4. In our study, the accuracy difference between the baseline and the bilateral condition was 9% whereas for Alvarez and Cavanagh it was 5%. Given the confidence intervals associated with these two measurements, these two values are consistent with each other. Similarly, the accuracy difference between the baseline and the unilateral conditions was also very similar for the two studies, in both cases being 26%. Thus, although the overall accuracy was slightly higher in the Alvarez and Cavanagh study, the relative accuracies of the three conditions were very similar in both studies, so we have successfully replicated their experiment.

The only difference between our results and those of Alvarez and Cavanagh [4] is that we found the difference between the bilateral and baseline conditions to be statistically significant, whereas Alvarez and Cavanagh did not. This difference appears to be due to our study having greater statistical power caused by our larger sample size (26 versus 8). Alvarez and Cavanagh also found accuracy to be less in the bilateral condition than in the baseline condition – they were just unable to prove that this difference was statistically significant.

## General Discussion

In this study we conducted a novel investigation into whether the identity-location bindings that are necessitated in tracking unique objects (MIT) would eliminate the bilateral tracking advantage that Alvarez and Cavanagh [4] reported when only locations needed to be tracked (MOT). We compared tracking performance for four targets located within one visual hemifield with tracking performance for four targets distributed between the left and right visual hemifields.

According to the logic of the paradigm, no difference in tracking performance between these two arrangements would indicate a non-hemifield-specific cognitive tracking resource. Conversely, higher accuracy for the bilateral arrangement would indicate a tracking resource that is *at least limited* in its capacity to be dynamically allocated across the visual field. Across four experiments our results show that the presence of identity-location bindings *reduces*, but does not entirely remove, the bilateral advantage (Experiments 1–3) relative to that observed when only locations need to be tracked (Experiment 4).

In Experiment 1, using a typical MIT display in which targets’ unique identities remained visible for the duration of movement, we found performance for the bilateral and unilateral arrangements to be unequal: tracking accuracy was greater when targets were divided between the left and right hemifields. In accordance with a set size effect [12], tracking accuracy for the baseline condition, in which observers tracked only two targets, was higher still. We ruled out the possibility that the bilateral advantage was a reflection of participants not maintaining fixation on the fixation cross in bilateral trials by replicating our finding using an eye-tracker.

In Experiment 2 we briefly cued the targets’ unique identities but kept them hidden for the remainder of the trial to ensure that observers had to continuously track the targets and could not ‘cheat’ by using the targets’ unique colours to recover their positions during the course of the trial. Still, we found a significant hemifield effect with tracking accuracy being again greater in the bilateral condition than in the unilateral condition.

In Experiment 3 we changed the number of targets to more closely reflect Alvarez and Cavanagh’s [4] original experiment (two unique targets and two distractors in each target quadrant). We found the same pattern of tracking performance as before and again observed a strong hemifield effect.

In Experiment 4 we conducted an MOT experiment, in which identity-location bindings were *not* required. This experiment was a close replication of Alvarez and Cavanagh’s [4] original experiment. We found a much stronger bilateral advantage than in our previous three MIT experiments. Unlike Alvarez and Cavanagh we found that accuracy in the baseline condition was significantly greater than that in the bilateral condition. This difference between the two studies seems to be due to our study having greater statistical power because our sample size was larger. Alvarez and Cavanagh also found tracking accuracy to be less in the bilateral condition than in the baseline condition, however for them the difference was not statistically significant. This finding is important because it shows that, even for MOT, tracking is not completely independent in the left and right visual hemifields. The fact that tracking accuracy is greater when tracking two targets in one hemifield (the baseline condition) than tracking two targets in each hemifield (the bilateral condition) shows that in the former condition resources from both hemispheres were used. Thus, our results show that tracking is only *partially* independent in the left and right visual hemifields and the degree of independence is greater for MOT than MIT.

MOMIT [24] is currently the only model of MIT. It proposes that identity-location bindings require a mechanism that serially cycles through each target, only ever attending to one target at a time. Every time a target is attended, its location is memorised. When it is time to re-attend to a given target the model assumes that whichever object is closest to the target’s previously remembered position is the target. The more targets there are, the longer it will take a given target to be re-attended, and hence the more chance that an error will occur. In this way, the model can explain why accuracy decreased with increasing set size, and hence why in all our experiments accuracy was greater in the baseline condition than in the other two conditions.

Because the model assumes that there is just a single attentive resource responsible for tracking all the objects in the visual field, it predicts no difference in tracking accuracy as a function of target arrangement. In particular, it predicts that tracking accuracy would be equal in the bilateral and unilateral conditions, contrary to the data of Experiment 1–3.

One could envisage a double serial model, in which there is a separate serial tracking mechanism for each hemifield. Assuming that the two tracking mechanisms operated independently, this could explain the bilateral advantage observed in our experiments. Unfortunately, this model would predict complete independence in tracking in the two hemifields so would predict that accuracy in the baseline condition would necessarily be equal to that in the bilateral condition. In none of our experiments was this observed.

Experiment 4 investigated an MOT stimulus. There have been a large number of models of MOT (for reviews see [6], [32]). For example, the FINST model of Pylyshyn and Storm [1] suggests that each target is tracked by a mental pointer known as a FINST. Because these FINSTs operate independently, the arrangement of the targets should not affect tracking accuracy. Thus, this model cannot explain why in Experiment 4 tracking accuracy was greater in the bilateral arrangement than in the unilateral arrangement.

The FLEX model of Alvarez and Franconeri [12] is similar to the FINST model in that it also assumes that each target is tracked by a mental pointer, although each pointer can be flexibly allocated a variable amount of a mental resource based on current tracking demands. The pointers are referred to as FLEX’s and each FLEX draws upon a common limited resource pool. The less resource the FLEX receives, the less able it is to track its target. The FLEX model assumes that there is a single, shared resource that all FLEXs draw upon, regardless of the arrangements of the targets. As such, the FLEX model must also incorrectly predict that tracking accuracy would be equal in the bilateral and unilateral arrangements in Experiment 4.

Franconeri and colleagues [14], [33] have proposed an alternative theory of MOT. According to their theory, tracking errors are primarily caused by spatial interference between targets, rather than any spatial interference between the targets and distractors, the speed at which the targets move, or the duration of movement. As long as there is adequate spacing between targets, they posit that we could theoretically track an unlimited number of them [33]. Of crucial relevance to the current study, spatial interference between two targets is assumed to occur less when they are in different hemifields than when they are in the same hemifield. This theory can accordingly explain why tracking is easier when targets are arranged bilaterally versus unilaterally.

Holcombe and Chen [34] have recently challenged the assumption that spatial interference between targets is the main cause of tracking errors. Their display had two pairs of disks that rotated about a common central axis, rather than moving freely. Each pair contained a single target. The inner pair moved on a circular track that had a radius of 2° and the outer pair moved on a circular track with a larger radius of either 4° or 9°. Tracking performance did not change with the radius of the outer pair, even though this caused the separation between the two targets to vary. Holcombe and Chen’s finding therefore challenges Franconeri and colleagues’ [33] proposition that tracking errors are always caused by spatial interference between the targets.

Battelli, Alvarez, Carlson, and Pascual-Leone [35] have proposed an alternative explanation for the bilateral advantage in object tracking. They used transcranial magnetic stimulation (TMS) to temporarily disrupt neural activity unilaterally in the intraparietal sulcus (IPS), an area that has been identified as strongly associated with object tracking in imaging studies [36], [37]. Tracking performance for targets in the visual hemifield contralateral to the site of the TMS interference decreased when targets were presented bilaterally. Conversely, when targets were presented unilaterally – regardless of whether they were ipsilateral or contralateral to the TMS disruption – there was no effect on tracking accuracy. The authors concluded that the left and right IPS have a contralateral tracking bias, but have the ability to track objects in both the contralateral and ipsilateral visual hemifields. Battelli and colleagues explained this as a process of mutual competition between the left and right IPS counterparts. According to this logic, the left and right IPS inhibit each other, so that each predominantly tracks objects in the contralateral hemifield. Interfering with IPS activity in one hemisphere necessarily removes its inhibitory influence on the other IPS. Provided that the targets are presented unilaterally, this thereby allows the uninhibited IPS to track targets in *either* the contralateral or ipsilateral visual hemifield. When targets are presented bilaterally, however, the uninhibited IPS will display its contralateral bias at the expense of the ipsilateral targets.

Battelli and colleagues’ [35] hypothesis can readily explain why it is easier to track objects bilaterally than unilaterally. It can also explain why tracking is not completely independent in the left and right hemifields: to some extent the IPS can track objects ipsilaterally. While this is a compelling hypothesis, it was developed only for MOT and needs to be extended before it can be applied to MIT and thus be able to explain all of our results.

All existing models of object tracking have been about *either* MOT (e.g. FLEX; [12]) *or* MIT (e.g. MOMIT; [24]). There are, however, obvious similarities between the two tasks: both require observers to sustain attention on multiple objects as their locations change. Indeed, Cohen and colleagues [22] have found evidence of a common resource for the location and identity aspects of the MIT task. They found that participants were able to *choose* to prioritise either the location or the identity aspect of an MIT task, and that prioritising one ultimately degraded performance on the other. This finding supports the idea that object tracking may be best conceptualised using a generalised model that addresses both MOT and MIT.

We therefore propose a working hypothesis for a combined model of MOT and MIT. We envisage a two-stage model, involving a first stage that segregates the targets from the distractors (i.e. MOT) and a second stage that associates a unique identity with each target (i.e. MIT). In the first stage, targets are segregated from distractors via multifocal attention, in a manner similar to that described by the FLEX model [12]. Unlike the original FLEX model, we assume that tracking operates separately in the left and right cerebral hemispheres, with each cerebral hemisphere in principle capable of tracking objects in both the left and right visual hemifields. In practice, due to competitive inhibition, each hemisphere is mainly (but not entirely) responsible for tracking targets in the contralateral visual hemifield, thereby creating the hemifield advantage observed in our experiments [35]. The first stage feeds the target locations to the second stage. The second stage builds on this information by continuously associating a unique identity with each target. We propose that this binding process is achieved by a central serial mechanism, shared between the two hemispheres, in a manner similar to that of MOMIT [24]. (The second stage differs from MOMIT in that it is explicitly informed of the target locations, so does not need to segregate the targets from the distractors.) Each target would be attended to in turn and its identity remembered. When it is time to reattend a given target, it would be assumed that whichever target is closest to that target’s previously remembered location is the target. If this is not the case, then an error will be made and the wrong identity would be associated with the wrong target.

Since the second stage builds upon the output of the first stage, it is entirely possible for the second stage to make an error even though the first stage performed correctly. If this were to happen, the observer would be able to successfully segregate the targets from the distractors, but would not be able to differentiate the targets from each other. In practice, this is a common occurrence [19].

This two stage model can also explain why hemifield effects are larger for MOT than MIT. When observers are asked to perform MOT there is no need to associate unique identities with the targets. Accordingly, the second stage is not engaged in the task, so cannot make any errors. All the errors thus result from the first stage leading to a large hemifield effect due to the strong contralateral bias of the IPS in the left and right hemispheres. Conversely, when the observer is asked to perform MIT, some of the errors would originate from the second stage. As these errors would not be affected by whether the targets are arranged bilaterally or unilaterally, due to the non-hemifield-specific nature of the serial mechanism, they will tend to make the performance in the bilateral and unilateral conditions more equal, thereby reducing the hemifield effects in MIT relative to MOT.

### Conclusions

In conclusion, in four experiments we have found evidence for partial, but not complete, hemifield independence in object tracking. In particular we found that tracking accuracy was greater when the targets were spread across both hemifields than when they were confined to a single hemifield. This hemifield advantage was greater in MOT, but was still significant in MIT. Our findings could be explained by a two-stage model of MIT, but the specific details of this theory will require further research.
